# Soft tissue chordoma of the right thigh demonstrated on ^18^F-FDG PET/CT and MRI: A case report

**DOI:** 10.1097/MD.0000000000047554

**Published:** 2026-02-13

**Authors:** Mengyue Liu, Jiaying Yu, Aijun Zhong, Zhongling Qiu, Juan Tang

**Affiliations:** a Department of Nuclear Medicine, Shanghai Sixth People’s Hospital, Shanghai Jiao Tong University School of Medicine, Shanghai, China; b Department of Critical Care Medicine, Weihai Hospital, Shandong University of Traditional Chinese Medicine, Weihai, Shandong, China; c Department of Pathology, Shanghai Sixth People’s Hospital, Shanghai Jiao Tong University School of Medicine, Shanghai, China.

**Keywords:** case report, chordoma, PET/CT, right thigh, soft tissue chordoma

## Abstract

**Rationale:**

Chordoma is a malignant bone tumor that typically affects structures along the midline. Soft tissue chordoma of the right thigh is an extremely rare entity but significant, as it can create diagnostic challenges and increases the risk of misdiagnosis, making accurate identification essential. This report introduces a diagnostic strategy of fluorine-18 fluorodeoxyglucose positron emission tomography/computed tomography (^18^F-FDG PET/CT) for a huge chordoma.

**Patient concerns:**

A 45-year-old man presented with a painless, gradually growing mass on his right thigh for 6 years without weight loss. Imaging studies revealed a huge mass in his soft tissue of the right thigh, raising concerns about pathology.

**Diagnoses:**

Magnetic resonance imaging demonstrated a mass with cystic mixed components, in the middle of the right thigh region with irregular shape and a poorly defined local boundary, measured 267 × 119 × 102 mm, leading to a presumptive diagnosis of malignancy.

**Interventions:**

^18^F-FDG PET/CT showed a huge area of heterogeneously increased ^18^F-FDG uptake (SUVmax, 4.4), and pathology and immunohistochemistry confirmed the diagnosis of conventional chordoma.

**Outcomes:**

He was treated with Imatinib Mesylate (400 mg) orally twice daily. There was no recurrence during the 6-month follow-up, with plans for continued long-term surveillance.

**Lessons:**

This case highlights the diagnostic challenge posed by chordoma of the soft tissue in the thigh, as its radiological appearance can closely resemble other soft tissue tumors. Clinicians should ensure exhaustive assessment including CT, magnetic resonance imaging, and ^18^F-FDG PET/CT imaging. Pathological confirmation is essential. Despite the rarity of soft tissue chordoma, careful treatment planning including consideration of targeted therapy and long-term follow-up is important to address the risk of late recurrence.

## 1. Introduction

Chordoma is a rare malignant bone tumor that consists approximately 2% to 4% of all primary malignant osseous tumors, commonly occurring in the sacrum and skull base, accounting for 55% and 30% of all the cases of chordomas, respectively.^[1,2]^ The high-risk population is patients older than 40 years, rarely affecting children.^[3,4]^ Chordoma is reported to be relatively benign characterized by asymptomatic and slow growth but can be locally aggressive due to its propensity for bone destruction.^[5]^ We report a patient presented with a mass in the soft tissue of the right thigh in magnetic resonance imaging (MRI) and fluorine-18 fluorodeoxyglucose positron emission tomography/computed tomography (^18^F-FDG PET/CT) images, and underwent a needle biopsy. Pathology confirmed the diagnosis of chordoma.

## 2. Case presentation

**Figure 1.**
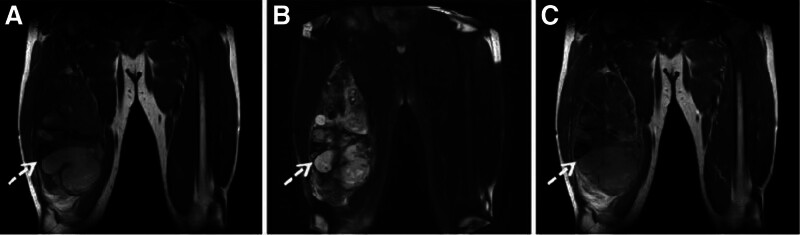
MRI revealed a huge space-occupying lesion with cystic mixed components, in the middle of the right thigh region with irregular shape and a poorly defined local boundary, measured 267 × 119 × 102 mm (white dashed arrow; A, T1-weighted coronal: T1 hypointense mass located over the middle of the right thigh region; B: T2-weighted coronal: T2 hypersignal and hypointense mixed; C: enhanced T1-weighted coronal: heterogeneous enhancement of the mass). MRI = magnetic resonance imaging.

**Figure 2.**
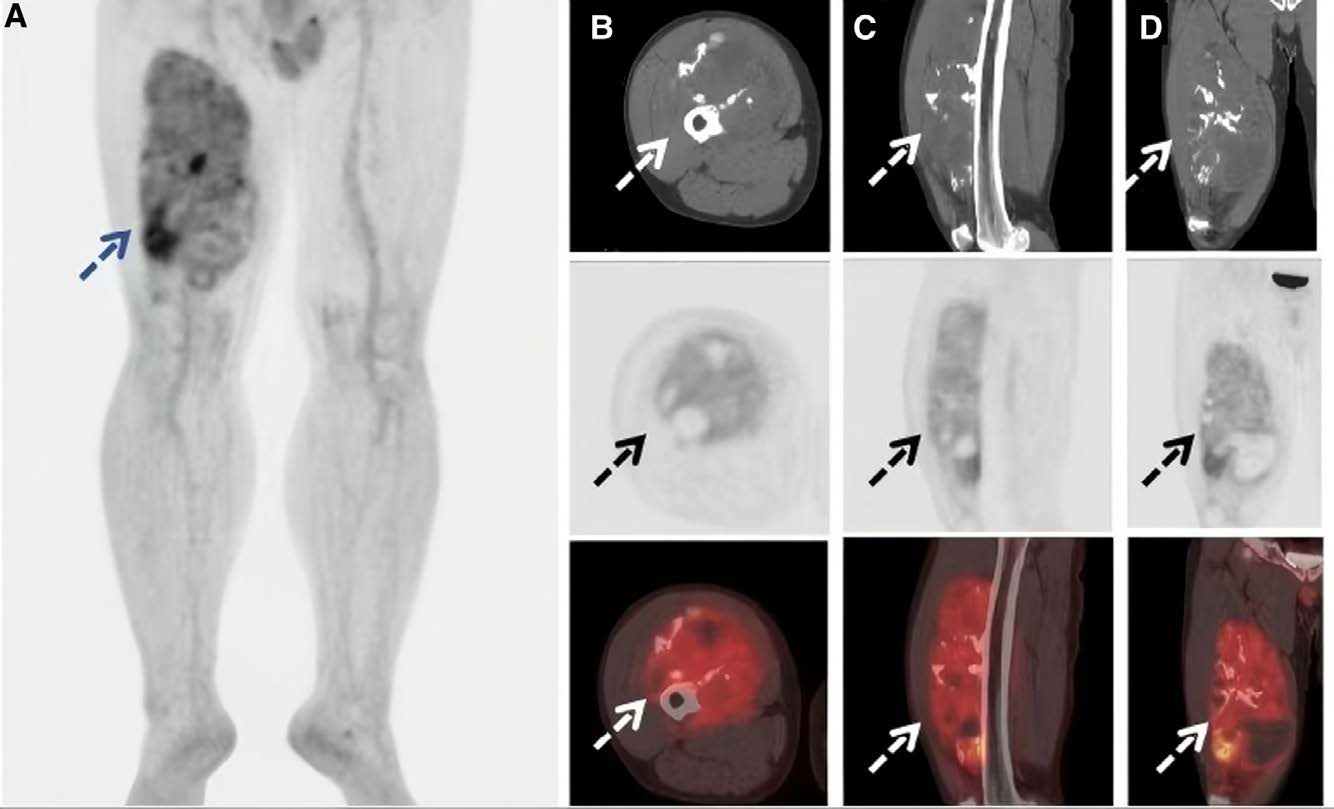
The whole-body MIP image (A) showed increased radiotracer uptake in the soft tissue of right thigh (blue dashed arrow). Right femur images showed a huge area of heterogeneously increased ^18^F-FDG uptake (SUVmax, 4.4) in the right thigh (white and black dashed arrow; B, axial; C, sagittal; D, coronal). 18F-FDG PET/CT = fluorine-18 fluorodeoxyglucose positron emission tomography/computed tomography, MIP = maximum intensity projection.

**Figure 3.**
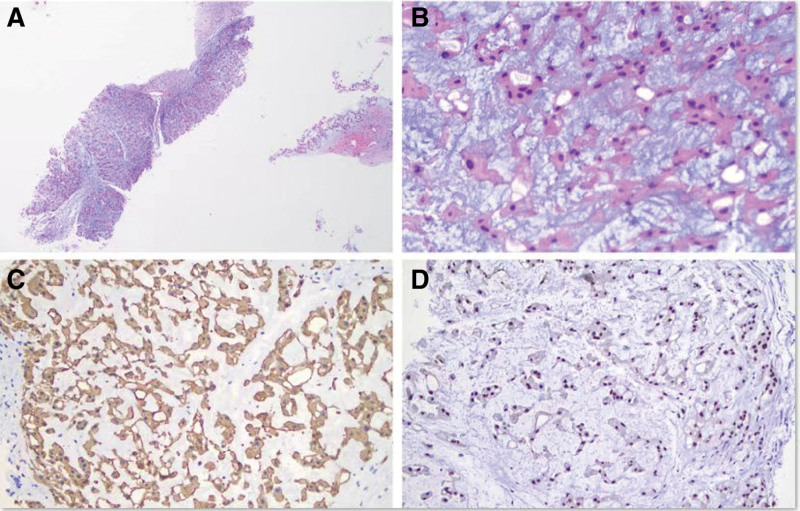
Tumor cells were arranged in sheets and cords separated by an abundant myxoid matrix (A, ×40; B, ×400). The tumor cells were diffusely positive for cytokeratin 19 (C) and brachyury (D) on immunohistochemistry, indicating that the tumor was extra-axial soft tissue chordoma.

A 45-year-old man presented to the outpatient department complaining of a palpable, slow-growing mass over the middle of the right thigh region for 6 years. The right thigh is slightly swollen. However, the patient denied a history of thigh trauma, fever, rash, weight loss, and lymphadenopathy. Urgent MRI was performed and coronal sections revealed a huge space-occupying lesion with cystic mixed components, in the middle of the right thigh region with irregular shape and a poorly defined local boundary, measured 267 × 119 × 102 mm (Fig. 1). Subsequently, the patient was scheduled for ^18^F-FDG PET. The whole-body maximum intensity projection image (Fig. 2A) showed increased radiotracer uptake in the soft tissue of right thigh (blue dashed arrow). Right femur images showed a huge area of heterogeneously increased ^18^F-FDG uptake (SUV_max_, 4.4) in the right thigh (Fig. 2B–D). Meanwhile, the patient quickly underwent a needle biopsy. Pathology and immunohistochemistry confirmed the diagnosis of conventional chordoma. Tumor cells were arranged in sheets and cords separated by an abundant myxoid matrix (Fig. 3A, ×40; B, ×400). The tumor cells were diffusely positive for cytokeratin 19 (Fig. 3C) and brachyury (Fig. 3D) on immunohistochemistry, indicating that the tumor was extra-axial soft tissue chordoma (STC).

## 3. Discussion

Chordoma, a rare tumor originating from remnants of the notochord, is a slow-growing malignancy that presents significant diagnostic and therapeutic challenges due to its tendency for local recurrence and resistance to standard treatments. Soft tissue chordoma is rarer than chordomas occurring at other anatomical locations. Usually, the chordoma has a direct effect through comprehension, leading to chronic pain. Additionally, due to their notochordal origin and distinct histological characteristics, the underlying mechanism may involve the dysregulated expression of proteins like cytokeratin, EMA, S100, and brachyury, which are frequently observed in chordomas.^[6]^ In this case, we discussed a 45-year-old male patient who presented with unclear pain and slight swelling in the thigh due to huge mass, which was later demonstrated as chordoma by histopathology examination.

Conventionally, CT and MRI are methods for assessment of the STC. In brief, ^18^F-FDG PET/CT, as a metabolic imaging modality, offers superior specificity, and is capable of identifying the occurrence of chordoma, differentiating metastases, and assessing treatment efficacy. Therefore, based on our experience, ^18^F-FDG PET/CT is recommended in the diagnosis and evaluation of patients with chordoma.^[7,8]^ As a subtype of axial chordomas, extra-axial chordomas can occur at rare sites distant from the middle, as reported in the literature, such as pontine, lung and nasopharyngeal.^[1,6,9]^ However, STC is exceedingly rare in clinical practice, particularly when it occurs in the thigh. In the past literature, STC of the thigh has been reported only twice, one of which detected by MRI while another without imaging information.^[3,10]^ To our knowledge, this is the 1st report of STC in the thigh detected by ^18^F-FDG PET/CT. Commonly, the thigh hosts soft tissue tumors, including benign lesions like lipomas, hemangiomas, and neurogenic tumors, as well as malignant ones such as liposarcomas and synovial sarcomas.^[11,12]^ Additionally, brachyury is a specific marker of chordoma, the diagnosis of chordoma has achieved consistency.

The STC is extremely rare, resection is performed and radiotherapy has been reported. However, there is no consensus on adjuvant chemotherapy in extra-axial STC.^[3]^ In the present case, we give the patient Imatinib Mesylate (400 mg) orally, twice daily.

Although the follow-up duration in this case is limited to 6 months, no evidence of local recurrence or metastasis was observed during this period. We acknowledge that chordoma has a well-known potential for late recurrence, sometimes even years after initial treatment. Therefore, we recognize this limitation and have planned continued regular clinical and imaging follow-up for this patient to ensure long-term surveillance.

Regarding the use of Imatinib Mesylate, although no universally accepted adjuvant therapy exists for extra-axial STC, this choice was based on its targeted inhibition of PDGFRB and c-Kit signaling pathways, which are implicated in chordoma biology.^[13]^ While molecular profiling was not performed in this case, previous studies have demonstrated such alterations in chordoma, providing a rationale for considering tyrosine kinase inhibitors in high-risk settings to reduce recurrence risk.

Future systemic-therapy considerations. Currently, no targeted therapies, cytotoxic chemotherapies or immunotherapies are formally approved for chordoma.^[13,14]^ TKIs such as imatinib, sunitinib and sorafenib have mainly been used in early-phase trials or under compassionate-use settings.^[14–16]^ However, single-agent TKIs offer only limited and temporary benefit, with reported median progression-free survival (PFS) typically 6 to 12 months, and resistance often developing after 2 to 3 months.^[15,17]^

Imatinib is generally well tolerated, with its safety profile mainly characterized in other indications such as GIST and CML. Common adverse events include fatigue, peripheral or periorbital edema, nausea, and mild rash, while ≥ grade 3 toxicities like hepatotoxicity and cytopenias are uncommon but require monitoring.^[18]^ Although chordoma-specific safety data are limited, similar adverse-event patterns are expected based on its pharmacological class.

Other TKIs such as dasatinib and sorafenib target overlapping pathways including PDGFR, VEGFR, and c-Kit. Dasatinib has been recommended in treatment guidelines and has shown a 54% PFS rate at 6 months in the SARC009 study of patients with unresectable chordoma.^[16,19]^ Sorafenib has demonstrated median PFS of 9 to 11 months in retrospective analyses, but can cause rash, diarrhea, fatigue, and hypertension.^[19]^ While head-to-head trials are lacking, these data suggest that alternative TKIs may offer comparable or modestly longer disease control with different toxicity profiles.

Taken together, these findings highlight the need for individualized treatment planning in chordoma, balancing molecular targets, comorbidities, patient preferences, and anticipated toxicities. Prospective trials and multicenter registries are needed to better define optimal sequencing, combination strategies, and integration with newer modalities such as immune checkpoint inhibitors.

### 3.1. Limitations

Despite providing valuable insights, this case report is limited by the relatively short 6-month follow-up and the single-case nature. Longer-term observation and additional research are essential to strengthen the evidence base and inform best practices for managing STC.

## 4. Conclusion

We report a rare case of STC of the right thigh diagnosed with MRI and ^18^F-FDG PET/CT imaging. This study confirms the value of ^18^F-FDG PET/CT in the diagnosis, assessment, and differential diagnosis of STC. Our case highlights the importance of advanced imaging, molecular profiling, targeted therapy considerations, and the need for long-term follow-up in managing this rare entity.

## Author contributions

**Conceptualization:** Zhongling Qiu, Juan Tang.

**Funding acquisition:** Zhongling Qiu.

**Writing – original draft:** Mengyue Liu, Jiaying Yu.

**Writing – review & editing:** Aijun Zhong, Zhongling Qiu, Juan Tang.
